# Evaluation of the Relationship Between Fracture Toughness and Hydrogen-Induced Damage in X70 Line Pipe Steel for Low-Temperature Service

**DOI:** 10.3390/ma19030552

**Published:** 2026-01-30

**Authors:** Reza Khatib Zadeh Davani, Enyinnaya George Ohaeri, Sandeep Yadav, Ehsan Entezari, Jerzy A. Szpunar, Michael J. Gaudet, Muhammad Rashid

**Affiliations:** 1 Department of Mechanical Engineering, College of Engineering, University of Saskatchewan, 57 Campus Drive, Saskatoon, SK S7N 5A9, Canada; 2 Research and Development, Interpro Pipe and Steel, 100 Armour Rd., Regina, SK S4K 0A8, Canada

**Keywords:** line pipe steel, fracture toughness, Electron Backscatter Diffraction (EBSD), X-ray diffraction (XRD), roughing and finishing (R/F) reduction, hydrogen induced cracking

## Abstract

In this study, X70 line pipe steels were subjected to different hot rolling treatments under three conditions with varying roughing (R) and finishing (F) reductions while maintaining the same total reduction to investigate the effect on drop weight tear test (DWTT) toughness and hydrogen-induced damage as assessed through electrochemical charging. Scanning Electron Microscope (SEM) images were used to analyze microstructure phases and their volume fractions, while Electron Backscatter Diffraction (EBSD) provided quantitative microscopy, and X-ray analysis examined crystallographic texture. Although all steels exhibited similar microstructure phases, the effective grain size and morphology varied slightly across the thickness. As these variations were minor, the focus shifted to other microstructural features such as textural characteristics. Overall, the steel with the medium R/F reduction demonstrated improved DWTT performance and greater hydrogen cracking and blistering resistance. This was attributed to stronger Transformed Brass (TBr) and Transformed Copper (TC) components, weaker Rotated-Cube (RC) texture, and lower Kernel Average Misorientation (KAM) values. Across the three steels in this work, this study demonstrates that increased fraction of blocky austenite/martensite as secondary phases, high geometrically necessary dislocation (GND) density, and RC texture negatively affect both DWTT and hydrogen damage resistance, whereas gamma (γ)-fiber and {332}<113> textures have positive effects. Improving these metallurgical factors can therefore boost toughness and reduce hydrogen-induced damage in line-pipe steels.

## 1. Introduction

With the global expansion of energy infrastructure, particularly in extreme environments, such as colder regions, there is a growing demand for line pipe steels that can perform reliably under low-temperature conditions. These line pipes play a critical role in the transportation of oil, gas, and other chemicals, making their mechanical integrity essential for preventing catastrophic failures. In cold regions, where temperatures often drop below freezing, line pipe steels must be designed to avoid brittle fracture, necessitating a high level of fracture toughness and mechanical resilience to meet stringent industry standards [1,2,3,4].

Line pipe performance is often evaluated through mechanical tests such as tensile testing, Charpy impact testing, and drop weight tear testing (DWTT). DWTT is particularly crucial for line pipe operating in low temperatures as it provides insight into a material’s resistance to a propagating brittle fracture and thus catastrophic failure [2]. Another significant challenge in maintaining the integrity of line pipe steels is the impact of hydrogen-related damage, including hydrogen-induced cracking (HIC) and hydrogen embrittlement (HE), both of which can severely degrade the mechanical properties of steels in low-temperature environments [5]. This consideration is especially important in the context of sour service line pipes, where exposure to hydrogen sulfide (H_2_S) can exacerbate hydrogen damage. As a result, the development of line pipe steels with enhanced DWTT performance and HE resistance is a key priority in the industry [2,6].

The thermomechanical controlled process (TMCP) is a highly effective technique for refining grain size and optimizing the overall structure of steel [7]. This process is especially useful for precisely controlling grain orientations and morphologies. This control can then be used to improve environmentally assisted degradation resistance in cold environments. Extensive research has been conducted on the influence of TMCP parameters on the properties of line pipe steel [8,9,10,11,12,13,14]. Roughing and finishing (R/F) rolling reductions are crucial TMCP parameters that significantly influence the microstructure, which subsequently impacts DWTT outcomes [15,16]. During the roughing rolling phase, higher reduction levels facilitate substantial grain refinement, breaking down larger grains and creating a more uniform and refined microstructure. This refinement enhances toughness by increasing grain boundaries, which act as obstacles to crack propagation, thus improving DWTT performance [15]. In the F rolling stage, additional deformation is introduced to further refine and align the microstructure, particularly affecting the grain size and texture. The F reduction is essential for promoting favorable texture components, like {332}〈113〉 and gamma (γ) fiber, which enhance DWTT results by improving the material’s ability to arrest cracks [17,18]. By carefully optimizing these parameters, it is possible to obtain a diverse range of steel microstructures, including ferrite, as well as minor constituents such as pearlite and bainite [11,15]. These tailored microstructures contribute significantly to the steel’s ability to withstand the challenging conditions associated with low-temperature service, thereby improving its overall performance and durability.

As the temperature decreases, the toughness of the steel diminishes until it reaches the ductile-to-brittle transition temperature (DBTT), at which point brittle fracture becomes more likely. Understanding and managing the fracture behavior of line pipe steel is critical for ensuring line pipe safety. This behavior is intrinsically linked to factors such as microstructure, crystallographic orientations, alloying elements, and precipitates [19,20]. Recent studies have shown that refining steel grains improves both the absorbed energy during low-temperature DWTT and the critical shear area percentage of 85% [21]. Grain refinement is often achieved by rolling the steel in the non-recrystallization region followed by rapid cooling, a process that enhances strength through the Hall–Petch relation [22,23]. Some research [24,25] has further demonstrated that smaller grains, obtained via ultrafast cooling, significantly contribute to increased strength and toughness in line pipe steel. A larger ferrite grain size generally increases the ductile-to-brittle transition temperature (DBTT) in pipeline steels, leading to a greater risk of brittle fracture at low temperatures. This is because larger grains provide longer, unobstructed paths for crack propagation [26]. Conversely, smaller grains provide more boundaries that can deflect and stop a crack from propagating, which requires more energy and shifts the DBTT to a lower value, improving toughness [27]. However, recent studies [28,29] showed an optimum range of grain size improved toughness at low temperature. In addition to grain size reduction, controlling grain orientation and phase composition could further enhance fracture toughness [24,30,31]. Additionally, variations in dislocation density introduced during TMCP can significantly affect both the strength and toughness, influencing DWTT outcomes [32]. Balancing dislocation density is therefore crucial to limiting crack initiation and propagation while improving mechanical performance at low temperatures. A higher dislocation density usually increases the steel’s yield and tensile strength. This happens because dislocations impede each other’s movement and make plastic deformation more difficult, thereby raising strength [33]. On the other hand, in DWTT, high dislocation density can contribute to crack initiation or propagation if the steel cannot accommodate further plastic deformation. While there is substantial evidence supporting the modification of processing conditions to achieve the desired microstructure or texture, achieving homogeneous grain orientation across the steel thickness continues to be a challenge in hot-rolled plates [34]. Duan et al. [35] found that a fine acicular ferrite, achieved through a fast cooling rate, resulted in higher DWTT absorbed energy and a lower DBTT. In contrast, coarse polygonal ferrite, produced through air cooling, exhibited lower absorbed energy and higher DBTT. Their investigation also revealed that the fracture surface with the highest fraction of grains oriented with the $\left\{001\right\}$ cleavage plane absorbed the least impact energy [35]. Moreover, when the per-pass reduction in the final roughing pass exceeded 15%, a fine, uniform microstructure of acicular ferrite was achieved, resulting in superior low-temperature fracture toughness [36]. These findings emphasize the need to refine grain size while carefully controlling grain orientation and secondary phases to optimize line pipe steel performance in low-temperature environments. While the effect of microstructure on mechanical properties has been widely studied, the influence of crystallographic texture and its key contributing factors remain underexplored.

Microstructure control has been reported as a critical factor in mitigating hydrogen-related degradation in line pipe steels. Previous studies show that a higher fraction of acicular ferrite and granular bainite, combined with the absence of segregation zones, can significantly enhance HIC resistance [37,38]. Additionally, recent studies [39] indicate that HIC tends to propagate more easily through grains with certain preferential orientations, such as $\langle 001\rangle ∥ND$, $\langle 113\rangle ∥ND$, and $\langle 112\rangle ∥ND$ (ND: Normal direction). Conversely, crystallographic orientations like $\langle 110\rangle ∥ND$, $\langle 111\rangle ∥ND$, and $\langle 332\rangle ∥ND$ have been shown to significantly improve HIC resistance by reducing intergranular crack paths [40,41]. This improvement is attributed to the increased number of coincidence site lattice (CSL) grain boundaries and low-angle grain boundaries (LAGBs) [9]. Non-metallic inclusions (NMIs) are crucial in both the initiation and growth of HIC due to their influence on localized stress fields and their hydrogen trapping capabilities [42,43]. The chemical composition of NMIs affects their interaction with hydrogen. In particular, oxide and sulfide inclusions have been observed within the crack paths of line pipe steels, indicating their contribution to crack propagation [8,44].

In this work, the influence of controlled thermomechanical rolling, through different combinations of roughing and finishing reductions, on the microstructure, texture, low-temperature toughness, and hydrogen-induced damage resistance of X70 line pipe steels was systematically investigated. While the steels exhibited similar phase constituents, subtle variations in grain size and morphology prompted a deeper examination of texture components, secondary phases, and GND-related features. The study aimed to identify which metallurgical factors most strongly govern DWTT performance and susceptibility to hydrogen cracking and blistering, and to determine how their optimization can enhance low-temperature serviceability. By integrating SEM, EBSD, and XRD analyses with quantitative evaluation of microstructural and textural contributions, this work provides a comprehensive understanding of the mechanisms that control toughness and hydrogen damage in line pipe steels and offers guidance for improved performance of pipeline steels. 

## 2. Materials and Methods

This study processed line pipe steels intended for sweet oil and gas to assess the impact of different roughing and finishing (R/F) deformations on the resulting microstructure, texture, and properties. The steels came from the same slab that was industrially cast (i.e., same chemistry), targeting an X70 grade as in Table 1 and was then subsequently pilot-scale rolled as presented in previous research [45] (From Interpro Pipe and Steel, Regina, SK, Canada). The three steel plates, part of the line pipe steel grade X70 group and designated as I-50, I-60, and I-70, shared the same microalloying composition as detailed in Table 1. In the sample codes, the letter “C” represents the centerline, while “Q” denotes the quarterline. Table 2 presents three distinct multi-pass rolling schedules designed to develop different textures with samples designated as I-50, I-60, I-70 by the approximate roughing reduction applied. Notably, I-70 steel had the highest roughing (R) reduction, while I-50 steel had the lowest. In contrast, finishing (F) reduction was highest in I-50 steel, medium in I-60, and lowest in I-70 steel. It is important to highlight that despite the variations in R/F reductions, all steels maintained a total thickness reduction of 90%. Other TMCP conditions (reheating, accelerated cooling, coiling simulations, etc.) were kept the same.

**Figure 1:**
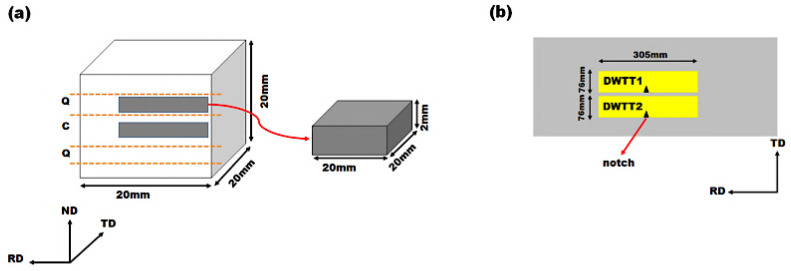
The schematic of (**a**) samples for microstructure analysis and microhardness test, and (**b**) DWTT samples extracted from the line pipe plate produced from pilot scale rolling. In (**a**), the orange dashed lines indicate the locations where the samples were cut, and the gray block represents the sample dimensions used for the investigations. (Q: quarterline, C: centerline).

Microstructure and texture evaluations were conducted on all line pipe steel specimens. Samples were prepared with dimensions of 20 mm (RD: Rolling direction) × 20 mm (TD: Transverse direction) × 2 mm (ND: Normal direction), machined from both the quarterline and centerline of the thickness (See Figure 1). In the sample codes, the letter “C” represents the centerline, while “Q” denotes the quarterline through the thickness of the steel. Microstructure and crystallographic texture of the studied steels were examined using a Hitachi SU6600 FESEM equipped with EBSD and connected to a computer running AZTEC 2.0 software (From Headquarters Hitachi High-Tech Canada, Toronto, ON, Canada). EBSD analysis was performed to characterize ferrite grain size, grain orientation, and texture components. The specimens were first prepared for microscopy and then subjected to additional polishing using a Buehler VibroMet 2 vibratory polisher with a 0.04 μm colloidal silica suspension (From Buehler in Lake Bluff, IL, USA) for 6–10 h following diamond polishing. After polishing, samples were rinsed with deionized water and ethanol, thoroughly dried, and mounted in a vacuum chamber for EBSD imaging. Measurements were conducted at an accelerating voltage of 30 kV with a step size of 0.14 μm over an area of 1200 × 900 μm^2^. The collected EBSD data was processed using Channel 5 software (Version 5.1, Oxford Instruments, High Wycombe, UK), recorded with AZTEC 2.0, and subsequently post-processed using HKL Project Manager software.

Drop weight tear testing is recognized for providing more accurate results compared to the conventional Charpy Impact test [46]. The DWTTs were conducted following API 5L [47] to evaluate the full-scale fracture response of each line pipe steel and the shear area percentage and absorbed energy were recorded. The DWTT specimens were full-thickness samples, measuring 12 inches (~305 mm) in length by 3 inches (~76 mm) in width, machined along the rolling direction with a notch oriented towards the transverse direction, according to Figure 1 schematic. According to the test standard [47], a minimum average shear area percentage of 85% is required for a test to be considered successful. Initial tests were conducted at −45 °C for all steel samples, followed by testing at −60 °C. To ensure reproducibility, a minimum of two samples were tested at each temperature.

The effect of hydrogen on the degradation of line pipe steels was evaluated using the electrochemical charging technique. For each steel, specimens were immersed in a single cell containing 0.2 M sulfuric acid and 3 g/L ammonium thiocyanate (Both from Sigma-Aldrich, Oakville, ON, Canada) at a current density of 20 mA/cm^2^. During electrolysis, hydrogen evolves at the steel surface and oxygen is generated at the platinum electrode. Ammonium thiocyanate promotes hydrogen entry by acting as a cathodic poison: SCN^−^ ions adsorb on the steel surface, suppress H_2_ bubble formation, increase the coverage of adsorbed hydrogen (H_ads), and facilitate its absorption into the lattice (H_ads_ → H_lattice_). Following hydrogen charging, specimens were rinsed with alcohol and analyzed for surface blisters and internal cracks. The total number of hydrogen-induced defects, along with the average crack length and blister area, were recorded for each sample. Specimens were then sectioned along the RD–ND plane to assess internal hydrogen-induced cracks, followed by polishing according to the previously described procedure. SEM imaging was subsequently used to evaluate the number and dimensions of internal cracks.

## 3. Results and Discussion

### 3.1. Analysis of Microstructural Features

#### 3.1.1. Microstructure Evaluation

**Figure 2:**
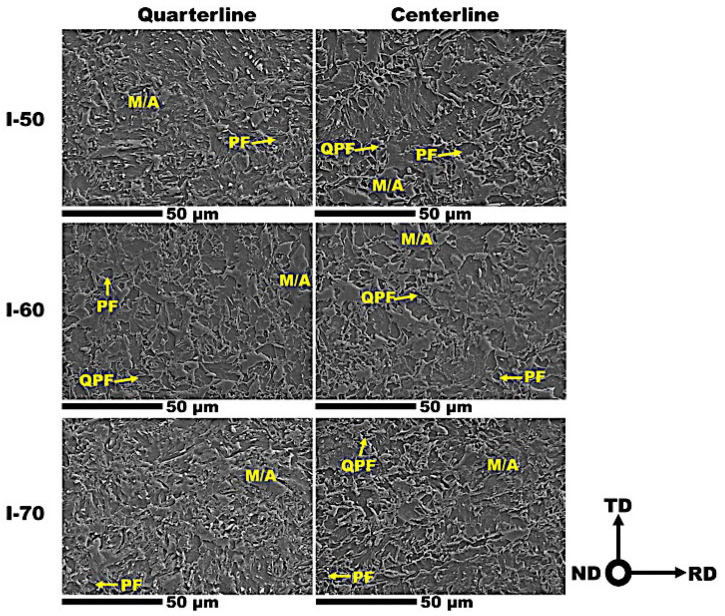
The microstructure micrographs of studied steels at two thickness layers in the RD-TD plane (RD: Rolling direction, TD: Transverse direction, PF: polygonal ferrite, QPF: quasi-polygonal ferrite, M/A: martensite/austenite island).

The SEM microstructures of the I-50, I-60 and I-70 processed steel sheets from RD-TD plane (RD: Rolling direction, TD: Transverse direction) at the quarterline (Q) and centerline (C) thickness are represented in Figure 2. The microstructure of all of these steel plates consists of polygonal ferrite (PF), quasi-polygonal ferrite (QPF), and martensite/austenite island (M/A) marked in the micro images. Although the general appearance of ferrite, bainite, and martensite is consistent with expectations for this steel grade, slight variations in grain morphology were observed across the steel thickness. Notably, the RD-TD plane exhibited a predominantly equiaxed grain morphology. The volume fraction of primary (PF, QPF) and secondary phases (bainite, M/A) was measured using ImageJ analysis software version 1.54k (From National Institutes of Health (NIH), Bethesda, MD, USA) in accordance with ASTM E562 [48], according to Table 3. The results indicate relative consistency across the studied steels, with values ranging from 10% to 13% (see Table 3). However, I-70 steel exhibited the highest volume fraction of secondary phases by ~13%.

#### 3.1.2. Quantitative Microstructure of Steels Using EBSD

**Figure 3:**
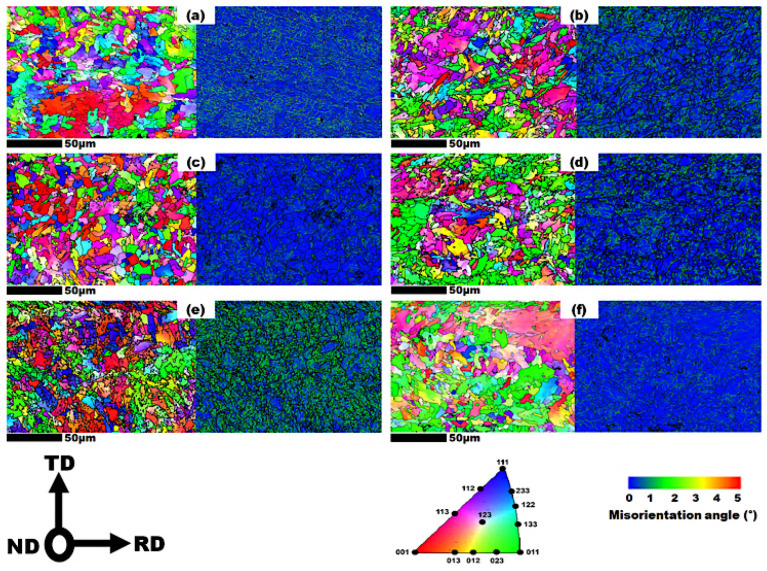
Grain orientation and KAM maps for RD-TD plane of (**a**) I-50Q (Q denotes as quarterline), (**b**) I-50C (C denotes as centerline), (**c**) I-60Q, (**d**) I-60C, (**e**) I-70Q, (**f**) I-70C (RD: Rolling direction, TD: Transverse direction, ND: Normal direction).

To analyze and quantify the microstructure and micro-texture of the steels, EBSD grain orientation maps were utilized, as shown in Figure 3. The average grain sizes for the quarterline and centerline thickness layers of the steels are summarized in Table 4. Among the steels, I-60 exhibited the largest average grain size at 6.8 µm, compared to 6.4 µm for I-50 and 4.7 µm for I-70. The quarter thickness region primarily contains refined grains, with some presence of coarse grains, as depicted in Figure 3. The grain size ratio of the quarterline to centerline thickness layer was 0.86, 0.90, and 0.88 for the I-50, I-60, and I-70 steels, respectively. These values indicate a homogeneous grain distribution across the thickness layers. It is noteworthy that while the grain size ratio is similar among the steels, I-60 steel exhibited the highest ratio, which may contribute to improved mechanical properties and reduced susceptibility to hydrogen-related damages. It was observed that in Figure 3, with an increase in R rolling reduction (from sample I-50 to I-70) in the quarterline, the texture intensities tended to move towards the $\left\{111\right\}$ and along with the reduction in $\left\{112\right\}$ orientations perpendicular to the ND. However, in the centerline layer, the $\left\{111\right\}$ and $\left\{112\right\}$ grain orientation intensity decreased, while the $\left\{100\right\}$ orientation intensity shifted towards $\left\{113\right\}$ and $\left\{012\right\}$ with increasing R reduction, as illustrated in Figure 3.

Furthermore, the kernel average misorientation (KAM) analysis, shown in Figure 3, measures the average misorientation between a reference point and its first nearest neighbor, derived from the EBSD scan maps. The KAM values for each steel and layer thickness are presented in Table 4. The KAM map provides an approximation of the dislocation density distribution by assessing the degree of orientation distortion within grains, particularly for misorientations less than 5°. According to Figure 3 and Table 4, the quarterline thickness generally exhibited lower KAM values compared to the centerline layer, suggesting a decrease in dislocation density across the thickness. However, this trend was reversed in the I-70 steel, where the quarterline layer showed a higher KAM value than the centerline layer. Overall, both I-50 and I-60 steels exhibited similar and lower KAM values compared to I-70 steel. This trend coincides with the increased R reduction, suggesting a potential correlation. Among the samples, I-60 steel had the lowest KAM value, while I-70 steel displayed the highest. This finding suggests that the relatively higher R reduction in the I-70 specimen correlates to a higher dislocation density, whereas the optimized combination of R/F reductions in the I-60 specimen led to the lowest dislocation density.

In this study, the geometrically necessary dislocation (GND) density was obtained from EBSD. The GND dislocation density value for studied steels is provided in Table 4. The determination of GND is particularly relevant for understanding plastic deformation and its influence on material properties. GNDs represent the dislocations required to accommodate lattice curvature within a material, arising from factors like grain boundary misorientation, plastic strain gradients, and geometrically constrained deformation [49]. While this calculation primarily reflects geometrically necessary dislocations, it provides a reliable overview of defect structures that can influence the mechanical properties and hydrogen damage susceptibility of line pipe steels. According to Table 4, the GND density aligns with the KAM values, and I-70 steel exhibited the highest value among the steels examined.

#### 3.1.3. Quantitative Microstructure of Steels Using X-Ray

**Figure 4:**
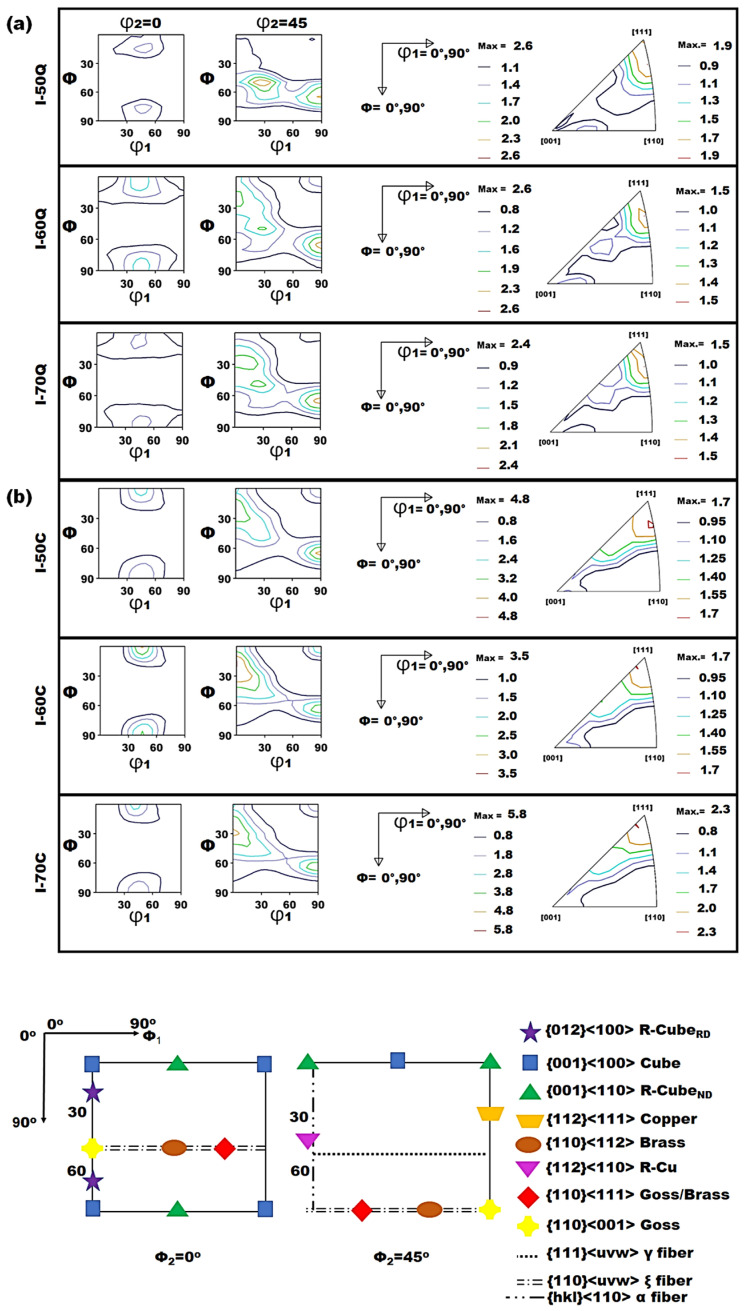
ODF φ_2_ sections for 0° and 45° and IPF maps for (**a**) quarterline, and (**b**) centerline thickness of steels.

X-ray diffraction (XRD) measurements were conducted to acquire statistical data on the texture distribution within each steel sample. The most significant texture components commonly observed in steels are represented on the φ_2_ = 0° and φ_2_ = 45° cross-sections of the orientation distribution function (ODF). The positions of these texture components are defined by the, φ_1_, φ and φ_2_ angles within the Euler angle space, with their locations detailed in the legend of Figure 4. Additionally, the corresponding Normal Direction (ND) Inverse Pole Figures (IPF) are illustrated in Figure 4. There was a consistent pattern in texture evolution across the thickness of the steels after undergoing various roughing/finishing (R/F) balances. The intensity maxima, identified at both thickness layers, were situated around similar positions within the Euler space for all samples. Although the overall texture in these steels may be considered weak, the ND IPF maps revealed a notable intensity shift towards $\left\{111\right\}$, with a minor spread towards the $\left\{001\right\}$ orientation perpendicular to the ND, as shown in Figure 4.

**Figure 5:**
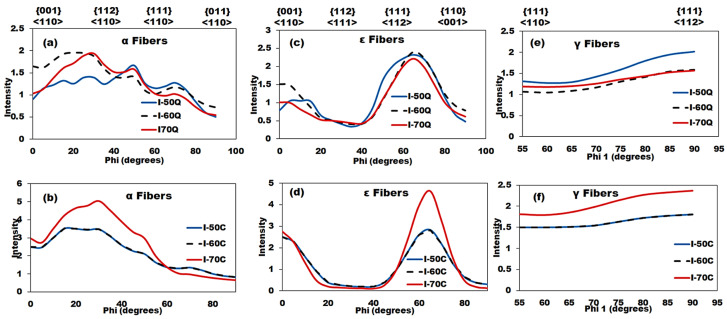
Orientation intensity plots for (**a**,**c**,**e**) quarterline thickness layer and, (**b**,**d**,**f**) centerline layer.

The primary texture components in the quarterline and centerline thickness layers of the line pipe steels are summarized in Table 5. The ODFs for the quarterline layer (see Figure 4a) predominantly exhibited Goss $\left\{110\right\}\langle 001\rangle$ texture intensity maxima, in contrast to the centerline, where other texture components, such as Copper, $\left\{332\right\}\langle 113\rangle$, Brass, Rotated-Cube (RC), and γ fiber, showed stronger intensities. These data are corroborated by the orientation intensity plots for both the quarterline and centerline, as presented in Figure 5. Notably, the I-50 and I-60 specimens displayed a higher intensity of the $\left\{332\right\}\langle 113\rangle$ texture component compared to the I-70 steel in both the quarterline and centerline layers. The increased $\left\{332\right\}\langle 113\rangle$ texture component in both the quarterline and centerline layers of I-50 and I-60 steels may be attributed to their higher finishing (F) rolling reduction compared to the I-70 steel. The maximum intensity of the $\left\{332\right\}\langle 113\rangle$ texture is likely due to austenite deformation occurring prior to its transformation into ferrite [50]. The typical texture component developed during the austenite deformation stage was the Cube $\left\{001\right\}\langle 110\rangle$, which eventually transformed into the RC $\left\{001\right\}\langle 110\rangle$ component. The recrystallization and subsequent deformation of austenite significantly influence the final microstructure and texture of dual-phase steels. While transformation below the critical temperature may not introduce new texture components, continued austenite deformation enhances existing ones. This effect strengthens the Transformed Copper (TC) texture component $\left\{112\right\}\langle 110\rangle$ due to lattice correspondence between austenite and ferrite [51], explaining its higher presence in I-70 steel (Table 5, Figure 5).

Additional texture fibers extracted from the ODFs, such as α $\langle 110\rangle ∥RD$, ε $\langle 110\rangle ∥TD$, and γ fibers $\langle 111\rangle ∥ND$, which represent the most critical grain orientations in hot-rolled line pipe steels, are presented in Figure 5. These orientations are often influenced by the applied processing conditions [52,53]. Furthermore, the I-50 steel exhibited a higher intensity of the γ fiber $\langle 111\rangle ∥ND$ in the quarterline compared to the other studied steels. In contrast, the highest γ fiber intensity in the centerline thickness was observed in the I-70 specimen. The γ fibers, along with the $\left\{332\right\}\langle 113\rangle$ and $\left\{554\right\}\langle 225\rangle$ texture components, are considered desirable in line pipe steels due to their association with enhanced mechanical properties, particularly strength and fracture toughness. Conversely, the RC texture component $\left\{001\right\}\langle 110\rangle$ is known to deteriorate fracture toughness [17]. According to Table 5, the I-70 specimen had the highest volume fraction of the RC texture component $\left\{001\right\}\langle 110\rangle$ within both thickness layers. This finding suggests that the austenite in I-70 steel underwent extensive recrystallization but limited non-recrystallization deformation before transformation, likely due to its high R reduction and low F reduction during rolling. As shown in Figure 5a, which also depicts the α fiber, all steels exhibited maximum texture intensities near the $\left\{112\right\}\langle 110\rangle$ and $\left\{223\right\}\langle 110\rangle$ orientations, with these intensities varying between the quarterline and centerline layers. Specifically, the I-60 steel showed the highest intensity of these orientations in the quarterline, while the I-70 steel demonstrated the maximum intensities in the centerline layer. The higher intensity of the RC $\left\{001\right\}\langle 110\rangle$ texture component in the centerline layer (see Table 4) indicates that more recrystallized austenite grains transformed into ferrite. Typically, the center layers contain more RC texture than the surface and quarterline layers.

### 3.2. Fracture Toughness Analysis

**Figure 6:**
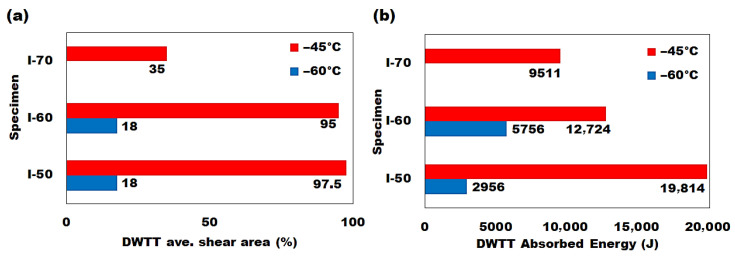
Fracture toughness response of line pipe steels (**a**) DWTT shear area percentage, (**b**) DWTT absorbed energy.

Fracture toughness is a critical property for line pipe steels, particularly in low-temperature applications, as it reflects the material’s ability to absorb energy prior to undergoing plastic deformation. According to the API 5L standard, the minimum shear area percentage required for a successful DWTT is 85% [47]. As shown in Table 6, only the I-50 and I-60 specimens met the DWTT criteria at −45 °C, with none of the steels achieving the required fracture toughness (85% shear area percentage) at −60 °C. The I-70 steel failed to pass the DWTT at −45 °C and was therefore not subjected to −60 °C testing. The DWTT results for line pipe steels processed under various roughing/finishing (R/F) conditions are illustrated in Figure 6. Among the steels tested, the I-50 steel absorbed the highest DWTT energy and exhibited a greater shear area on its fracture surface after testing at −45 °C. This result suggests that the higher finishing (F) reduction employed in processing I-50 steel may have contributed to its enhanced fracture toughness. This observation implies that a lower roughing (R) deformation followed by a higher finishing (F) reduction is beneficial for improving low-temperature fracture toughness. The grain sizes of I-50 and I-60 steels were close; however, the slightly smaller grain size of I-50 may contribute to its higher DWTT energy at −45 °C. As the grain size of the I-60 specimen was larger than that of the I-50 steel (Figure 3), this large grain structure may have contributed to the higher DWTT absorbed energy observed for the I-60 steel at −60 °C (Figure 6) because larger grains reduce grain boundary density, which lowers obstacles to dislocation movement and crack propagation [35,54].

The contradictory impact of grain size on DWTT indicates that it is not the only factor influencing DWTT results in line pipe steels. Also, recent research has shown that although an increased number of reduction steps generally results in smaller grain sizes, varying levels of rolling reduction did not significantly alter the grain size [55]. In addition, the dislocation density has a substantial impact on DWTT performance. According to Table 3, I-70 steel showed the highest dislocation density, which corresponded to the lowest DWTT results. A higher GND density generally signifies a more complex dislocation structure, thereby increasing brittleness and reducing toughness through the buildup of dislocations that impede crack propagation [49].

Texture components, in addition to grain size, have an impact on DWTT. Some texture components contribute significantly to the toughness of line pipe steels. A study on ferritic stainless steel revealed that different rolling reduction sequences, including 67% + 50% and 50% + 67% routes did not significantly affect grain size but did influence texture development and anisotropy [56]. The previous work showed that while the microstructure features of steels are similar, the minor differences in crystallographic texture affect the mechanical properties, particularly the DWTT of line pipe steels [45]. According to Table 5, the higher intensities of the $\left\{332\right\}\langle 113\rangle$, γ fiber, and TC texture components observed in the I-50 and I-60 specimens are reflected in their superior DWTT absorbed energy compared to the I-70 specimen. In contrast, the I-70 steel exhibited a higher RC texture component, which is known to deteriorate mechanical properties, particularly fracture toughness. Also, the I-50 and I-60 steels exhibited higher Goss texture components in both the quarterline and centerline layers compared to the I-70 steel, which may be related to their improved fracture toughness in low-temperature applications. Additionally, in I-70, the presence of the $\left\{100\right\}$ crystallographic cleavage plane, oriented parallel to the fracture surface, has been identified as a factor that can reduce the toughness of line pipe steel [2,45]. The KAM value also has a significant impact on DWTT performance. A higher KAM suggests greater misorientation within the grains, which can promote microcrack formation and reduce the material’s ability to absorb energy during fracture [57]. According to Table 4, I-70 steel exhibited the highest KAM value, which helps explain its lower toughness in DWTT. Although the microstructures of the investigated line pipe steels were not significantly different, it is evident that minor variations in microstructure and crystallographic texture have a measurable impact on their mechanical properties. Thus, it is important to minimize microstructural and texture inhomogeneity and control the development of favorable grain orientations by adopting an appropriate TMCP strategy for steel processing [58,59].

### 3.3. Hydrogen Damage

#### 3.3.1. Analysis of Hydrogen Blisters

**Figure 7:**
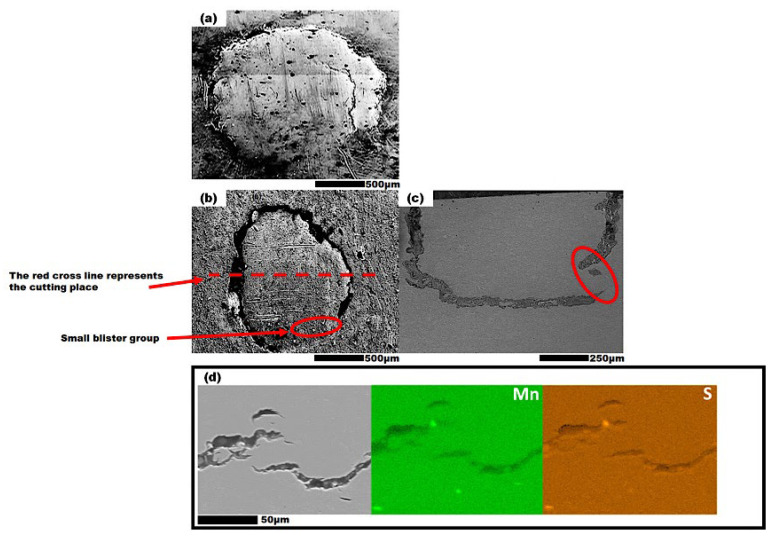
(**a**) The blister on the surface of I-50 specimen, (**b**) the blister on I-70 specimen, (**c**) the cross section of the blister in I-70 specimen, (**d**) high magnification and the EDS analysis of the crack initiation/path in the blister in part (**b**,**c**).

Number density, average diameter, and area of the blisters observed in the I-50, I-60, and I-70 steel plates are given in Table 7. The I-50 steel showcased larger blister diameters and less number density in comparison to I-70 steel, leading to less surface area damage. The I-60 steel did not exhibit any blistering on its surface. Based on the information in Table 4 and Table 5, the susceptibility of steels to hydrogen blistering is influenced by their microstructure and texture characteristics. The I-60 steel, with its larger grain size and higher fraction of TC, Transformed Brass (TBr), and $\left\{332\right\}\langle 113\rangle$ textures, showed better hydrogen blistering resistance than I-70 steel, which had a higher RC texture fraction, leading to more blisters. Two typical blisters observed on the surfaces of I-50 and I-70 steels are represented in Figure 7. The blistering patterns were notably different between the two steels. On the I-50 steel surface (Figure 7a), tiny cracks were visible at the base of the hydrogen blister. In contrast, some blisters on the I-70 steel surface exhibited a “blister on blister” pattern, where additional blisters formed atop existing ones (Figure 7b). This pattern often emerges when hydrogen charging exceeds one hour [60]. Eventually, rupture occurs when the blister breaks through the surface (Figure 7a). Figure 7c shows the cross-section of the blister from Figure 7b, revealing several cracks in the top layer of the steel, consistent with the internal pressure theory explaining blister formation.

The Energy Dispersive X-Ray Spectroscopy (EDS) map analysis was conducted on the blister in the I-70 steel to identify the elements present around the nucleation sites, as shown in Figure 7. The most common elements associated with non-metallic inclusions were selected for scanning, including Fe, Mn, S, Si, Ca, Ti, Cr, and O. Upon completing the scan, only Mn and S showed distinct sharp points in the EDS map (marked by red circle), indicating that the inclusion along the crack path was MnS. To enhance the clarity and readability of the image, other elements were excluded from the final presentation as they did not exhibit sharp points or contribute to hydrogen trapping at the site. It is worth noting that there may be a possibility of CaO forming a complex NMI with MnS. However, the signals for Ca and O were not sufficiently sharp or distinct in the map. Repeated analyses failed to confirm their presence as a clear component of a complex NMI.

#### 3.3.2. Analysis of Hydrogen-Induced Cracking

**Figure 8:**
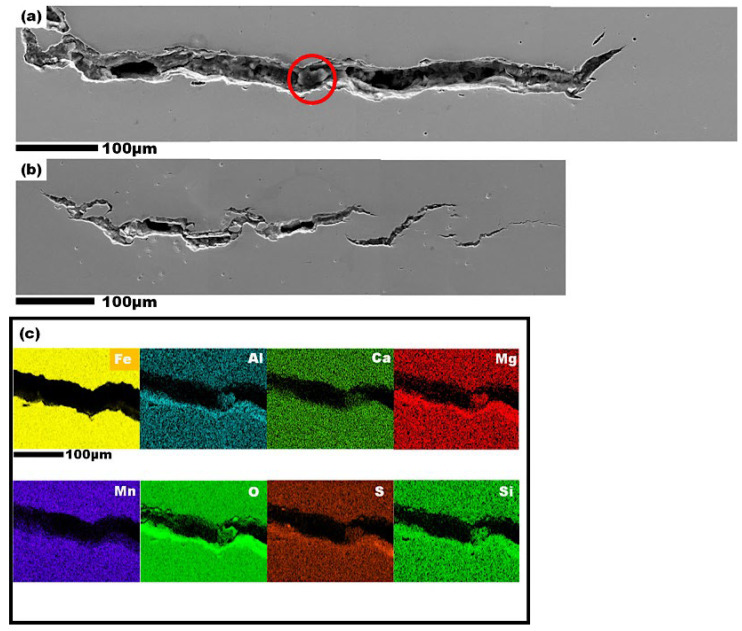
(**a**) Continuous hydrogen crack of I-70 steel, (**b**) disconnected hydrogen crack in I-70 steel, (**c**) the EDS maps of the crack path (marked by red circle in part (**a**)) in I-70 specimen.

The susceptibility of the steels to HIC was only observed in the I-70 steel after 24 h of hydrogen charging, with no evidence of HIC in the I-50 and I-60 steel samples at the same charging time. In Figure 8, two significant cracks, measuring a total of 850 µm, were visible in the quarterline and centerline thickness layers of the I-70 steel, suggesting that HIC propagates in a stepwise, linear, and disordered manner (Figure 8a,b). The absence of blisters and cracks in the I-60 steel sample indicates a higher hydrogen attack resistance compared to the other steels. This improved behavior may be attributed to differences in crystallographic texture and grain boundary characteristics. Recall that the I-60 steel exhibited the lowest KAM value (Figure 3), which correlates with its lower susceptibility to hydrogen damage, in contrast to the I-70 steel, which exhibited a higher KAM value, indicative of a higher dislocation density, which is likely responsible for its lower HIC resistance among the studied steels. The measured dislocation density of I-70 in Table 4 was the highest among the steels, confirming this statement. Moreover, I-60 steel displayed stronger TC $\left\{112\right\}\langle 110\rangle$ and TBr $\left\{111\right\}\langle 112\rangle$ texture components compared to I-70 steel, which had a higher fraction of RC, potentially diminishing its HIC resistance. Previous studies [9] have suggested that a strong $\langle 001\rangle ∥ND$ grain orientation in line pipe steel is detrimental to HIC resistance, while $\langle 111\rangle ∥ND$ and $\langle 112\rangle ∥ND$ texture components are more effective in preventing HIC propagation.

**Figure 9:**
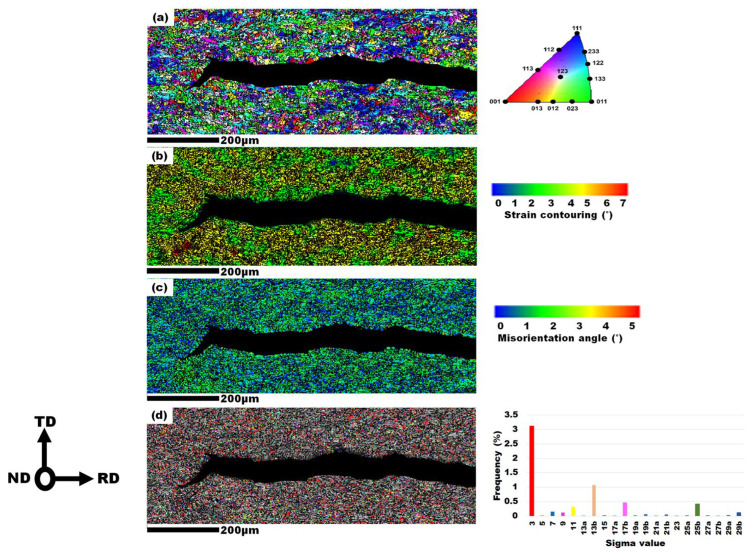
The EBSD analysis on a HIC site at the cross-section of the mid-thickness layer (ND-RD) of I-70 specimen (**a**) grain orientation map, (**b**) strain contouring map, (**c**) KAM map, (**d**) CSL boundaries map.

The EDS spectra of HIC taken from the selected area of the crack marked by the red circle is shown in Figure 9c. According to the EDS maps, non-metallic inclusions, including MgO, Al_2_O_3_, SiO_2_, and MnS, were evident within the crack path. Most hydrogen cracks were initiated from non-metallic inclusions, such as oxides and sulfides, rather than from nitrides and carbides. Nitrides and carbides often have relatively small sizes (less than 1 μm, on average), while oxide and sulfide inclusions typically have diameters of 2–3 μm. Thus, inclusions like Al_2_O_3_ can attract more hydrogen than nitride inclusions, resulting in the formation of cracks beside them [38,61]. Similarly, a recent study [62] claimed that MnS-Si inclusions were found along the steel matrix’s transgranular and intergranular crack paths.

LAGBs are defined as grain boundaries with a misorientation angle of less than 15°, while HAGBs have a misorientation angle greater than 15° [63]. The low angle grain boundary (LAGB) and high angle grain boundary (HAGB) percentages in studied steel thickness layers displayed in Table 8, based on EBSD analysis, showed an average of 24% HAGB and 76% LAGB. There is ongoing controversy regarding the role of LAGBs and HAGBs in crack growth and hydrogen damage susceptibility. Some researchers argue that HAGBs facilitate crack propagation more than LAGBs, due to their higher atomic disorder and hydrogen trapping [64]. When hydrogen accumulates at the trapping sites, it can cause localized increases in internal pressure and stress, weakening the cohesion at the grain boundary and making it more susceptible to cracking [65]. On the other hand, others suggest that low-angle grain boundaries (LAGBs) reduce hydrogen crack growth rate by acting as barriers to dislocation motion, as detailed in the research by Tavenner et al. [66] and Entezari et al. [67]. In this study, the effects of HAGBs and LAGBs on hydrogen damages were not distinct because the percentage of these grain boundaries was consistent across all steels. Consequently, no evidence of their specific impact on hydrogen damage susceptibility could be established in the studied steels.

The grain orientation, strain countering, KAM, and coincidence site lattice (CSL) boundary maps from EBSD analysis of intergranular HIC in the mid-thickness layer of I-70 steel are presented in Figure 9. According to Figure 9a, the crack propagated predominantly along random grain orientations, aligning with previous findings [55]; however, Venegas et al. [39] and Masoumi et al. [68] reported that HIC typically propagates along the $\langle 100\rangle ∥ND$ cleavage planes. As shown in Figure 9b, the high-strain zones (red-yellow) near the crack tip and along the crack path were associated with significant plastic deformation due to dislocation pile-up and interactions with hydrogen atoms, suggesting crack initiation sites [69]. The KAM map (Figure 9c) highlights areas with high local misorientation density (depicted by a green-yellow gradient), which act as stress concentrators, supporting the crack growth [70,71].

Further EBSD analysis in Figure 9d shows the CSL boundary map for the crack tip area, where the proportion of Σ3 grain boundary types was the highest, while the Σ5 grain boundary was less prevalent. According to Mohtadi Bonab et al. [72], there is a higher frequency of Σ3-type boundaries near the HIC, which is consistent with the findings in Figure 9d. Arafin et al. [73] reported that several CSL boundaries, such as Σ11 and Σ5, play an important role in the arrest of cracks; however, these observations were made in systems where intergranular cracking dominated the failure process.

### 3.4. Connection Between DWTT and Hydrogen Damage Resistance

**Figure 10:**
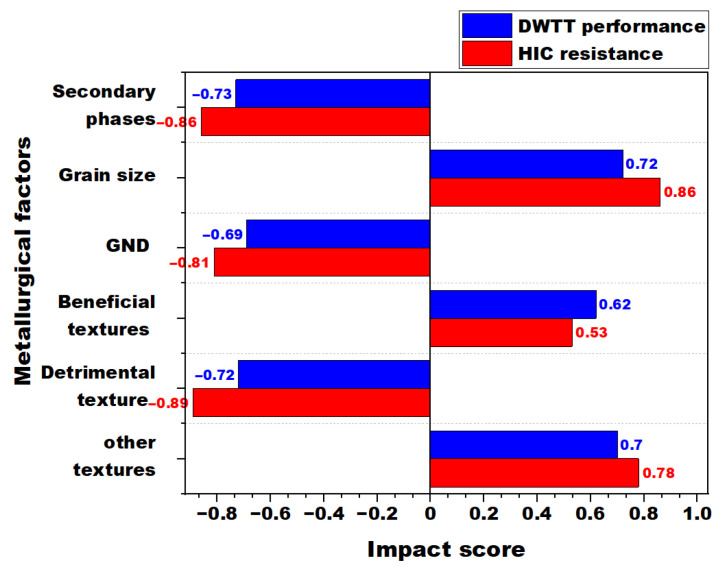
The relative significance of metallurgical factors, indicated by impact score, on HIC resistance and DWTT performance of steels (metallurgical factors include secondary phase volume fraction, grain size, geometrically necessary dislocation (GND) density, beneficial textures: {332}〈113〉 texture component and γ fiber, detrimental texture: RC texture component, and other textures including TBr, TC, and Goss texture components).

A quantitative approach was employed to assess the impact of various metallurgical factors on the HIC resistance and DWTT performance of line pipe steels. Three samples, designated as I-50, I-60, and I-70, were analyzed by normalizing all metallurgical parameters, including secondary phase volume fraction, grain size, GND density, and texture components. Additionally, the measured HIC crack length and DWTT energy were normalized to ensure consistency in comparison. The impact score for each factor was then determined to establish their relative influence on the studied variables, according to Figure 10. This method allows for a systematic evaluation of the role of microstructural and textural features in defining the mechanical properties and HIC resistance of line pipe steels. This impact score, ranging from −1 to +1, effectively represents the standardized effect of each factor, enabling a direct comparison of their relative importance. A score closer to +1 indicates a strong positive influence, suggesting that an increase in the parameter leads to improved performance (higher DWTT energy or HIC resistance). Conversely, a score closer to −1 reflects a strong negative influence, implying that an increase in the parameter leads to diminished performance (lower DWTT energy or HIC resistance). This approach allows for a comprehensive assessment of the complex interplay between various metallurgical factors and the mechanical properties and HIC resistance of line pipe steels.

The findings in Figure 10 indicate that microstructural attributes, including secondary phase volume fraction, grain size, and GND density, have a similar significant impact on both properties. Specifically, secondary phase volume fraction and GND density have a strong negative impact on DWTT performance and HIC resistance, with higher impact scores for HIC resistance, ranging from −0.7 to −0.86. The increased volume fraction of secondary phases and higher GND densities contribute to localized stress concentrations, promoting crack initiation and propagation, thereby reducing impact toughness and increasing susceptibility to hydrogen-induced damage [69,74]. According to Figure 10, in addition to microstructural features, texture components play a critical role in determining mechanical performance and HIC resistance. Beneficial texture components, including γ fiber and {332}<113> texture component, exhibit a positive impact on DWTT and HIC resistance, as reflected in their impact scores. In contrast, the RC texture component, identified as a detrimental texture, had a significant negative effect on DWTT performance and a strong negative correlation with HIC resistance, showing a higher impact score magnitude compared to beneficial textures. Other texture components, including TBr, TC, and Goss texture components, demonstrate a moderate influence on both variables but are not the dominant factors governing mechanical behavior and hydrogen damage susceptibility in line pipe steels. These findings align with the results reported by Jing et al. [75], who also observed that secondary phases and GND density negatively affect DWTT performance, while beneficial texture components contribute to improved toughness and HIC resistance. Although microstructural features such as grain size and phase composition significantly influence the studied variables, it is essential to recognize that texture components play an equally critical role in determining the mechanical properties and HIC resistance of line pipe steels. In conclusion, the metallurgical factors were found to have a consistent trend in their effects on both DWTT performance and HIC susceptibility, indicating a direct relationship between these two variables. The results suggest that optimizing metallurgical factors similarly enhances both DWTT performance and HIC resistance in the studied line pipe steels.

## 4. Conclusions

The microstructure and crystallographic texture of line pipe steels, cast industrially and processed using TMCP at a pilot scale facility with varying roughing (R) and finishing (F) reductions for low-temperature applications, were thoroughly investigated. The analysis of fracture toughness in low-temperature tests was conducted by examining differences in microstructure, texture, and grain size. Additionally, the susceptibility of the steels to hydrogen-induced damages was assessed through electrochemical charging. Based on these analyses, it can be concluded that I-60 steel with medium R and F rolling reduction had the best DWTT performance, as well as hydrogen induced damage resistance. The quantitative analysis of metallurgical factors on DWTT performance and HIC resistance in the studied steels emphasized the significant role of texture components on low-temperature mechanical properties and susceptibility to hydrogen-induced damages. Based on the analyses, the following conclusions can be drawn:

- The microstructure of the steels in RD-TD plane include ferrite, and martensite/austenite islands. The microstructure of the studied steels was quite similar, with a comparable secondary phase volume fraction and a quarterline-to-centerline grain ratio close to 0.9, indicating a relatively homogeneous microstructure throughout the thickness. In all steels, the grain size at the centerline thickness layer was larger than at the quarterline. Among the studied steels, I-60, which underwent medium R/F reduction, had the largest grain size, measuring 6.8 µm.
- Although the crystallographic texture was weak in all steels, with some preference for $\left\{111\right\}$ and $\left\{100\right\}$ plane orientations perpendicular to the ND, the centerline thickness showed a stronger texture intensity compared to the quarterline thickness. There was a consistent increase in the volume fraction of the major texture components, including γ fiber, Transformed Brass (TBr), Transformed Copper (TC), and $\left\{332\right\}\langle 113\rangle$ texture components in the centerline layer of the steels.
- For low-temperature applications, steels I-50 and I-60 performed better in DWTTs than steel I-70. This suggests that the higher F reduction in I-50 and I-60 resulted in a higher fraction of beneficial texture components like $\left\{332\right\}\langle 113\rangle$ which likely contributed to the improved toughness.
- I-70 steel with the highest R reduction, had the largest area of blister and the highest crack length. In contrast, I-60 steel, with medium R/F reduction, demonstrated the highest hydrogen-induced blisters and crack resistance due to its favorable textural characteristics.
- The impact score analysis revealed that secondary phase volume fraction, GND density, and RC texture exerted the strongest negative influence on both DWTT toughness and HIC resistance, while γ-fiber and {332}<113> textures had clear positive effects. This consistent behavior across parameters demonstrates a direct link between microstructural/textural optimization and improved both low-temperature fracture and hydrogen-induced degradation resistance.

## Figures and Tables

**Table 1:** Chemical composition (wt. %) of X70 line pipe steels (adapted from [45]).

C	Mn	Cu + Ni + Cr + Mo	V + Nb + Ti
<0.05	1.3	<0.95	0.07–0.08

**Table 2:** Thermomechanical rolling schedule of X70 line pipe steels.

Sample	Total Roughing Reduction (%)	Total Finishing Reduction (%)
I-50	43–53	78–88
I-60	55–65	68–78
I-70	67–77	62–72

**Table 3:** Volume fraction of secondary phases (%) in the studied steels. (Q: Quarterline thickness layer, C: Centerline thickness layer).

Steel	Q	C	Total Average
I50	10.35 ± 1.1	10.81 ± 0.5	10.58
I60	9.79 ± 0.4	10.28 ± 0.6	10.04
I70	11.44 ± 0.5	12.77 ± 1.0	12.1

**Table 4:** The grain size and KAM value of steels at different thickness layers obtained from EBSD maps (Q: Quarterline thickness layer, C: Centerline thickness layer).

	Grain Size (µm) ± 0.2			KAM Value			Dislocation Density × 10^15^ (m^−2^)		
Steel	Q	C	Total Average	Q	C	Total Average	Q	C	Total Average
I-50	5.9	6.8	6.4	0.08	0.14	0.11	6.91	12.09	9.5
I-60	6.4	7.2	6.8	0.08	0.14	0.11	6.91	12.09	9.5
I-70	4.4	5.0	4.7	0.29	0.08	0.19	25.05	6.91	16.41

**Table 5:** Volume fraction of major texture components at quarterline (Q) and centerline (C) layers of steels (TC: Transformed Copper, TBr: Transformed Brass, RC: Rotated Cube).

Samples	{332}<113>	TC-I	TC-II	TBr	RC	γ Fiber	Goss Fiber	Max Intensity
I-50Q	8.6	8.2	8.9	11.7	3.1	17.2	1.9	3.5
I-50C	12.7	13.7	15.1	18.3	4.1	19.7	2.2	5.2
I-60Q	8.2	8.9	10.1	13.3	3.2	15.6	1.8	2.6
I-60C	10.7	12.9	14	16.6	4.5	17.5	1.4	4.1
I-70Q	7.1	9	9.4	10.2	3.4	15.8	1.1	2.7
I-70C	10.2	12.1	13.3	15.6	4.8	22.8	0.7	5.7

**Table 6:** DWTT performance of steels at two different temperatures.

Steel	DWTT Performance	
	−45 °C	−60 °C
I-50	Pass	Fail
I-60	Pass	Fail
I-70	Fail	-

**Table 7:** Statistical distribution of observed hydrogen-assisted blisters on the surface of studied steels.

Steel	Number Density of Blisters	Average Blister Diameter (µm)	Total Blister Area (mm^2^)
I-50	3	1247	1.64
I-60	-	-	-
I-70	7	911	2.84

**Table 8:** LAGB and HAGB fraction percentage of steels.

Steel	LAGB Fraction (%)	HAGB Fraction (%)
I-50 Q	82	18
I-50 C	69	31
I-50 (total average)	76	24
I-60 Q	81	19
I-60 C	70	30
160 (total average)	76	24
I-70 Q	82	18
I-70 C	70	30
I-70 (total average)	76	24

## Data Availability

The original contributions presented in this study are included in the article. Further inquiries can be directed to the corresponding authors.
